# Absence of SARS-CoV-2 in Second-Trimester Amniotic Fluid During the SARS-CoV-2 Virus Pandemic

**DOI:** 10.1177/26884844251387022

**Published:** 2025-10-09

**Authors:** Poonam Samyal, Luis A. Bracero, Andrew Quinn, Ying Chan

**Affiliations:** ^1^ Comprehensive Perinatal Medicine, PLLC, Flushing, New York, USA.; ^2^ Department of Obstetrics and Gynecology, South Shore University Hospital, Bay Shore, New York, USA.; ^3^ Zucker School of Medicine at Hofstra/Northwell, New Hyde Park, New York, USA.; ^4^ Department of Obstetrics and Gynecology, Long Island Jewish Medical Center, New Hyde Park, New York, USA.

**Keywords:** amniocentesis, vertical transmission, coronavirus, COVID-19, obstetrics, maternal morbidity, perinatal outcomes, nucleocapsid antibodies, Spike IgG

## Abstract

**Objective:**

To determine the presence or absence of severe acute respiratory syndrome coronavirus 2 (SARS-CoV-2) in second-trimester amniotic fluid during the SARS-CoV-2 virus pandemic in patients undergoing genetic amniocentesis.

**Study Design:**

Women undergoing amniocentesis between September 2020 and April 2022 were asked to participate in the study. On the day of amniocentesis, an additional 5 mL of amniotic fluid, 5 mL of maternal blood sample, and nasal swabs were collected from each participant. None of the patients had active SARS-CoV-2 infection on the date of amniocentesis. Demographic data and history of SARS-CoV-2 virus infection were collected. After 2021, the SARS-CoV-2 virus vaccination status was obtained. Amniotic fluid and maternal nasal swabs were collected and tested for the presence of the SARS-CoV-2 virus *via* real-time reverse-transcriptase polymerase chain reaction test using an automated bead-based method for extracting total nucleic acid. An electrochemiluminescence immunoassay on maternal blood was used to detect SARS-CoV-2 total (immunoglobin G/immunoglobin M) antibodies against nucleocapsids.

**Results:**

This study enrolled 92 patients. There were 16 patients who reported a history of SARS-CoV-2 virus infection: 8 of them reported SARS-CoV-2 virus infection during the first and second trimesters and 8 reported SARS-CoV-2 virus infection before pregnancy. A total of 49 patients were vaccinated prior to amniocentesis. All 92 amniotic fluid samples tested negative for the SARS-CoV-2 virus. There were five positive and three negative SARS-CoV-2 maternal nucleocapsid antibody tests among the eight patients who had the SARS-COV-2 viral infection during the first and second trimesters of pregnancy. All eight patients had negative swab tests for the SARS-CoV-2 virus.

**Conclusions:**

There was no SARS-CoV-2 virus in the second-trimester amniotic fluid samples from the eight patients who reported SARS-CoV-2 viral infection during the first and second trimesters. This finding suggests that, after a first- and second-trimester SARS-CoV-2 virus infection, it is unlikely to find the SARS-CoV-2 virus in the amniotic fluid.

## Introduction

The first cases of pneumonia caused by severe acute respiratory syndrome coronavirus 2 (SARS-CoV-2) were documented in December 2019 in Wuhan, located in Hubei Province, China. Recognizing its severity and widespread impact, the World Health Organization (WHO) officially designated this novel coronavirus infection as “Coronavirus Disease 2019” (COVID-19) and declared it a global pandemic on March 11, 2020.^1,2^ Since the COVID-19 pandemic, our understanding of SARS-CoV-2 infections and immunity has increased significantly. A scientific brief published by the WHO in February 2021 discussed three distinct mechanisms regarding the vertical transmission of SARS-CoV-2. These mechanisms include (1) intrauterine transmission, whereby the virus is transmitted during pregnancy through the placenta; (2) intrapartum transmission, which occurs during labor and childbirth through contact with vaginal secretions, maternal blood, or fecal matter; and, finally, (3) postnatal transmission, which can occur through contact with the infected mother, such as breastfeeding.^3^

It is debatable whether SARS-CoV-2 can be transmitted by the mother to the fetus. The vertical transmission rate of SARS-CoV-2 is inconclusive and fluctuates between 0% and 3.3%.^4–7^ Most of the data on vertical transmission are based on cases of COVID-19 that occurred in the late second or third trimester of pregnancy,^8^ while data on vertical transmission following first- and second-trimester infection are scarce. The purpose of our study was to determine whether the SARS-CoV-2 virus can be found in the amniotic fluid of women with first- and second-trimester SARS-CoV-2 viral infections undergoing second-trimester genetic amniocentesis.

## Methods

All patients undergoing genetic amniocentesis between September 2020 and April 2022 were eligible for this study. Amniocentesis was performed at Comprehensive Perinatal Medicine PLLC, New York, United States, and Northwell Health Physician Partners Maternal Fetal Medicine at Bay Shore offices, New York, United States. The study protocol and informed consent were approved by the Northwell Health Institutional Review Board, 20-0588. All participants signed an informed written consent.

Demographic data and history of SARS-CoV-2 virus infection were collected using a patient questionnaire. SARS-CoV-2 virus vaccination status was added to the questionnaire after July 2021. On the day of the procedure, an additional 5 mL of amniotic fluid was collected. Five milliliters of maternal blood and nasal swab specimens were also collected from each participant.

The maternal nasal swabs and amniotic fluid samples were tested for the presence of the SARS-CoV-2 virus using real-time reverse-transcriptase polymerase chain reaction (RT-PCR) for amplification, and an automated bead-based method was used for isolating and purifying SARS-CoV-2 total nucleic acid. To perform the COVID-19 RT-PCR test, SARS-CoV-2 nucleic acid was first extracted, isolated, and purified from nasal swabs and amniotic fluid samples using the MagMAX Total RNA Isolation Kit (Thermo Fisher Scientific) on the KingFisher Flex instrument (Thermo Fisher Scientific, Waltham, MA). The purified nucleic acid was then reverse-transcribed into complementary DNA, followed by PCR amplification and detection using the QuantStudio 7 Flex RT-PCR instrument (Thermo Fisher Scientific). An electrochemiluminescence immunoassay was used on the maternal serum to identify SARS-CoV-2 total (immunoglobin G [IgG]/immunoglobin M) antibodies against nucleocapsids. A positive result suggests an immune response to a recent or prior history of COVID-19. This test would not be expected to detect an immune response to a messenger RNA (mRNA) vaccine. Spike IgG antibodies against SARS-CoV-2 spike proteins were also assayed, where a positive result indicates an immune response to mRNA vaccination or to a prior infection with SARS-CoV-2. Testing for SARS-CoV-2 viral infection was not performed in placental tissue, umbilical cord blood, and newborns. Medical records were subsequently reviewed to gather information such as maternal demographics, gestational age at delivery, route of delivery, birth weight of the infants, and vaccines received.

## Results

We enrolled 92 patients who had genetic amniocentesis during the COVID-19 pandemic. The participants were enrolled between September 2020 and April 2022. All patients were asymptomatic, with no evidence of active COVID-19 infection at the time of genetic amniocentesis. The mean maternal age was 33.7 ± 6 years, with a mean gestational age at amniocentesis of 18.5 ± 2 weeks.

Delivery data and route of delivery were available for 76 participants (Table 1). The mean gestational age at delivery was 37.3 ± 4 weeks, and the mean neonatal birth weight was 3.0 ± 0.7 kg. There were 71 live births. Out of the 76 patients, 61 (80.3%) of these women delivered full term and 48 (63.2%) of them delivered vaginally. There was one second-trimester pregnancy loss 1 week after the genetic amniocentesis. This fetus had echogenic bowel and bowel obstruction diagnosed at 20 weeks. It was believed that she had intrauterine infection since the amniotic fluid retrieved was dark and foul smelling. Spontaneous rupture of membranes occurred at 21 weeks and she delivered shortly upon presentation to labor and delivery. The nasal swab result was negative for SARS-CoV-2 virus, and nucleocapsid total antibodies were also negative in this patient. Therefore, this loss is not likely to be caused by the SARS-CoV-2 virus. In addition to this pregnancy loss after amniocentesis, there were four terminations: three for fetal abnormalities and one for fetal demise *in utero*. Pregnancy outcomes were unknown in 16 cases.

**Table 1.** Delivery Data and Route of Delivery for 76 Participants

Mean gestational age at delivery	37.3 ± 4 weeks
Mean neonatal birth weight	3.0 ± 0.7 kg
Term live births	61 (80.3%)
Preterm live births	10 (13.2%)
Second-trimester pregnancy loss shortly after amniocentesis	1 (1.3%)
Termination of pregnancy	4 (5.3%)
Route of delivery	
Vaginal deliveries	48 (63.2%)
Cesarean sections	28 (36.8%)
Unknown	16

Indications for amniocentesis were abnormal maternal serum screening/positive noninvasive prenatal testing (21), advanced maternal age (26), fetal congenital abnormalities (13), soft markers on ultrasound examination (15), positive genetic carrier screening test results (7), previous pregnancies with congenital anomalies (5), fetal demise (1), family histories of genetic abnormalities (2), and history of infection/drug exposure in the first trimester (2). One patient had gastritis and *Helicobacter pylori* infection; she received multiple medications prior to finding out that she was pregnant. This patient was apprehensive and requested amniocentesis. A second patient had possible toxoplasmosis in the first trimester and opted for amniocentesis. None of these two patients reported a history of SARS-CoV-2 infection.

Among 92 amniotic fluid samples tested, all of them were negative for the SARS-CoV-2 virus. The SARS-CoV-2 nucleocapsid antibody test was performed on 76 patients, with 16 patients’ specimens not being analyzed due to hemolysis during the collection and transit process. Among the 76 patients who were tested for SARS-CoV-2 nucleocapsid antibodies, 20 (26%) tested positive and 56 (74%) tested negative. Among those 20 patients who tested positive for nucleocapsid antibodies, 18 of them also tested positive for spike antibodies. The spike antibodies were not analyzed on the remaining two patients due to hemolysis of the blood sample. The antibodies against the spike protein can be present either after natural infection or vaccination, whereas antibodies against the nucleocapsid protein are only present following natural infection.

There were 16 patients who reported a history of COVID-19 in this study: 8 of them had SARS-CoV-2 virus infection during the first or second trimesters and 8 contracted SARS-CoV-2 virus infection before pregnancy. None of the 16 patients who had COVID-19 required hospitalization, and they all reported having only a mild SARS-CoV-2 viral infection. All of them had negative nasal swab tests for the SARS-CoV-2 virus antigen at the time of genetic amniocentesis. Among the eight patients who reported SARS-CoV-2 during the first and second trimesters of pregnancy, there were five positive and three negative maternal SARS-CoV-2 nucleocapsid antibody tests.

A total of 49 patients were vaccinated prior to amniocentesis. Twenty-four of them received Moderna (Cambridge, MA), 19 received Pfizer-BioNTech (Mainz, Germany), 3 received Johnson & Johnson (Leiden, Netherlands), 2 received SinoVac (otherwise known as Beijing Kexing Bioproducts, Beijing, China), and 1 received Sinopharm/Vero Cell (Beijing, China). Among the vaccinated patients, 30 patients (91%) tested positive and 3 (9%) tested negative in the Spike IgG antibody test. The blood samples of 16 patients were rejected by the laboratory, as their quality was compromised due to the same reason as stated above.

Pregnancy outcomes and laboratory data among eight participants who reported COVID-19 in the first and second trimesters are described in Table 2. Delivery information was available for six patients. Case 1 delivered spontaneously 1 week after amniocentesis for presumptive intrauterine infection at 21 weeks. Three out of six patients were term deliveries and two were preterm deliveries. Delivery outcomes were not available on two patients. The five patients with positive SARS-CoV-2 maternal total nucleocapsid antibodies also tested positive for Spike IgG antibodies.

**Table 2.** Clinical Characteristics and Laboratory Data in Eight Patients Who Reported Severe Acute Respiratory Syndrome Coronavirus 2 Infection in the First and Second Trimesters

Case No.	SARS-CoV-2 vaccination	GA at COVID-19 infection	GA at delivery	BW (kg)	Amnio results	Nasal swab (RT-PCR) results	Nucleocapsid total (IgG/IgM) antibodies	Spike IgG
1	No	6 weeks	21 weeks	N/A	46 XY FISH NL Microarray NL	N	N	N/A
2	Yes	6 weeks	N/A	N/A	46 XY Microarray NL	N	N	N/A
3	Yes	8 weeks	39 weeks	3.23	46 XX Microarray NL	N	P	P
4	Yes	15 weeks	39 weeks	2.86	46 XX Microarray NL Combination of single alpha Hgb gene deletion and Hgb Constant Spring	N	N	N/A
5	Yes	18 weeks	39 weks	2.63	46 XX Abnormal Microarray: Female with a Shox gene deletion	N	P	P
6	Yes	6 weeks	34 weeks	1.79	46 XX Microarray NL	N	P	P
7	Yes	8 weeks	N/A	N/A	47 XY Trisomy 21	N	P	P
8	Yes	17 weeks	34 weeks	2.00	46 XY Microarray NL	N	P	P

BW, birth weight; GA, gestational age in weeks; Hgb, hemoglobin; IgG, immunoglobin G; IgM, immunoglobin M; N, negative; NL, normal; N/A, not available; P, positive; RT-PCR, real-time reverse-transcriptase polymerase chain reaction; SARS-CoV-2, severe acute respiratory syndrome coronavirus 2; FISH, Fluorescence In Situ Hybridization.

## Discussion

This is a case series involving asymptomatic women having a genetic amniocentesis during the COVID-19 pandemic. Notably, all amniotic fluid samples tested negative for the presence of the SARS-CoV-2 virus.

Most of the studies on the vertical transmission of SARS-CoV-2 have concentrated on symptomatic infection in the third trimester,^8,9^ with the majority being case series and retrospective studies.^10,11^ Miscarriage, preterm delivery, fetal growth restriction, and fetal death have been reported to be increased under the condition of maternal SARS-CoV-2 infection in the third trimester.^12–14^ However, the effects of SARS-CoV-2 infection in the first and early second trimester on the pregnancy and the fetus remain unclear.

Rosen et al.^15^ performed amniocentesis on 22 patients who had COVID-19 infection in the first and second trimesters of pregnancy; all 22 amniotic fluid SARS-CoV-2 RT-PCR tests were negative. Lorente et al. reported two cases of pregnant women with COVID-19 infection during the first and second trimesters; both cases were negative for SARS-CoV-2 in amniotic fluid.^16^ The patients in Rosen’s study were likely to have higher SARS-CoV-2 viral load since they all had laboratory-proven infection based on nasal and pharyngeal specimens. In the eight patients in our study who reported having COVID-19 during the first and second trimesters, no SARS-CoV-2 virus was detected in the amniotic fluid samples. This finding suggests that it is highly unlikely to find the SARS-CoV-2 virus in the amniotic fluid, after a first- and second-trimester SARS-CoV-2 virus infection. Our findings align with previous studies indicating a lack of evidence for perinatal SARS-CoV-2 infection among infants born to mothers who experienced SARS-CoV-2 infection during pregnancy.^17,18^

There are several limitations to this study. One limitation is that the patients self-reported having the SARS-CoV-2 viral infection, which may not be accurate. Three out of eight women (37%) who reported having SARS-CoV-2 infection during pregnancy had negative nucleocapsid antibodies, this can be attributed to inaccurate self-reporting or false-negative nucleocapsid antibody test results. In addition, pregnancy is known to be an immunocompromised state in order to prevent rejection of a fetus as a foreign body. Therefore, nucleocapsid antibodies may not appear in some SARS-CoV-2-infected pregnant women. Another limitation is that none of the patients reported having severe SARS-CoV-2 viral infections. An additional limitation is the lack of SARS-CoV-2 testing data from placentas, umbilical cord bloods, and newborns. Urine and amniotic fluid are known to have the lowest rate of SARS-CoV-2 detection when compared to blood, placental, and nasopharyngeal specimens. We did not study vertical transmission, as testing for SARS-CoV-2 infection was not performed on the placenta, umbilical cord blood, and neonates. Kotlyar et al. have reported SARS-CoV-2 positivity to be 2% *via* neonatal nasopharyngeal swab, 2.9% *via* cord blood, 7.7% *via* placental samples, 0% *via* amniotic fluid, 0% *via* urine samples, and 9.7% *via* fecal or rectal swab.^19^ Urine and amniotic fluid have the lowest yield, as it is believed that SARS-CoV-2 infects the placenta first, then passes through the gastrointestinal tract, and is consequently identified in fecal specimens.

Among the 20 participants with nucleocapsid antibodies, half of them reported a history of COVID-19. Therefore, asymptomatic SARS-CoV-2 infection was present in at least 10 other patients from September 2020 to April 2022. In addition, 18 out of the 20 patients were tested for spike antibodies, and all 18 patients were positive for spike antibodies. The high concordant rate between the presence of nucleocapsid and spike antibodies is usually observed in patients who have a history of COVID-19.

While vertical transmission of SARS-CoV-2 was not addressed in this study because of lack of placental and neonatal testing, our findings are relevant and reassuring for women who are infected with SARS-CoV-2 in pregnancy. Amniocentesis is widely used in pregnant women to diagnose a variety of congenital viral infections such as cytomegalovirus, parvovirus B19, and varicella. However, diagnosing congenital infection associated with SARS-CoV-2 *via* amniocentesis should be avoided in view of the reported absence of virus in the amniotic fluid.

Although the WHO declared the end of COVID-19 as a public health emergency on May 5, 2023, many pregnant women continue to be diagnosed with COVID-19 infections. Our data can serve to reassure pregnant women that the SARS-CoV-2 virus is not likely to be found in amniotic fluid after a first- and second-trimester mild SARS-CoV-2 viral infection.

This work was presented in a poster entitled “Absence of SARS-CoV-2 in Second Trimester Amniotic Fluid during the SARS-CoV-2 Virus Pandemic: A Prospective Study” at the Society of Maternal Fetal Medicine’s 44th Annual Meeting at National Harbor, Maryland, USA, in February 2024.^20^

## Data Availability

The data supporting the conclusions of this article will be made available by the authors upon reasonable request.

## Abbreviations Used

BW: birth weight
COVID-19: Coronavirus Disease 2019
FISH: Fluorescence In Situ Hybridization
GA: gestational age in weeks
Hgb: hemoglobin
IgG: immunoglobin G
IgM: immunoglobin M
mRNA: messenger ribonucleic acid
N: negative
N/A: not available
NL: normal
P: positive
RT-PCR: real-time reverse-transcriptase polymerase chain reaction
SARS-CoV-2: severe acute respiratory syndrome coronavirus 2
WHO: World Health Organization
